# Nexrutine and exercise similarly prevent high grade prostate tumors in transgenic mouse model

**DOI:** 10.1371/journal.pone.0226187

**Published:** 2019-12-19

**Authors:** Darpan I. Patel, Kira Abuchowski, Roble Bedolla, Paul Rivas, Nicolas Musi, Robert Reddick, A. Pratap Kumar

**Affiliations:** 1 School of Nursing, UT Health San Antonio, San Antonio, Texas, United States of America; 2 Mays Cancer Center at UT Health San Antonio, San Antonio, Texas, United States of America; 3 Sam and Ann Barshop Institute for Longevity and Aging Studies, UT Health San Antonio, San Antonio, Texas, United States of America; 4 Department of Urology, School of Medicine, UT Health San Antonio, San Antonio, Texas, United States of America; 5 Department of Pathology, School of Medicine, UT Health San Antonio, San Antonio, Texas, United States of America; University of Minnesota Twin Cities, UNITED STATES

## Abstract

The purpose of this investigation was to compare the antitumorigenic effects of the natural product Nexrutine to voluntary wheel running (VWR) in the transgenic adenocarcinoma of the mouse prostate (TRAMP) model. Forty-five, 10-week old TRAMP mice were randomized to either receive free access to the running wheel, Nexrutine pelleted into chow at 600 mg/kg or no treatment control. Mice were serially sacrificed at weeks 4, 8,12 and 20 weeks. Palpable tumors, body weight, food consumption and running wheel activity were monitored weekly. At necropsy, tumors and serum were harvested and stored for analysis. Serum was used to quantify circulating cytokines in 4 and 20 week time points. Nexrutine supplementation led to a 66% protection against high grade tumors. Exercise resulted in a 60% protection against high grade tumors. Both interventions reduced concentrations of IL-1α. Exercise also significantly lowered concentrations of eotaxin, IL-5, IL-12(p40) and VEGF. While there were no significant differences at baseline, exercise mice had significantly lower IL-5 and VEGF compared to control at the 20 week time point. Nexrutine also significantly reduced circulating IL-9 concentrations. No significant differences were observed when compared to the control group. Immunohistochemistry of tumor sections showed significantly lower expression of pAkt in Nexrutine fed mice with no visible differences for NFκB. In conclusion, both Nexrutine and exercise suppressed tumor growth. Though similar outcomes were seen in this comparative effectiveness study, the mechanisms by which exercise and Nexrutine exert this benefit may focus on different pathways.

## Introduction

Prostate cancer (PCa) is the most common solid-tumor cancer in American males and is the second most common cause of cancer death [1]. Development of PCa spans several decades with clinical detection of PCa manifesting in patient in their 60s [2]. This long latency period provides a significant opportunity to implement chemopreventative interventions to inhibit the transition from pre-neoplastic lesions to malignant cells. It has been reported that cancer risk reduces significantly with an active lifestyle and a diet rich with phytochemicals [3, 4]. Lack of healthy activity and diets in the US point to increased incidence rates of several cancers, including PCa, compared to other countries [5].

Exercise has been shown to be beneficial in men with PCa [6–12]. However, the impact of exercise on tumor physiology are not clearly understood. A recent systematic review by Shephard found mixed results on the benefits of physical activity on PCa prevention with 45 of the 83 trials reviewed presenting diminished risk [13]. Prospective cohort studies have also found that lifelong physical activity is directly related to reduced risk of developing high grade tumors upon biopsy [14–16]. In men diagnosed with PCa, physical activity is associated with lower overall mortality and PCa mortality [17] and reduced risk of recurrence [18, 19]. Though deemed beneficial, the mechanisms by which exercise confer these benefits remain understudied.

Studying the effects of exercise on cancer prevention are difficult and time-consuming requiring years of interventional data. For that reason, relying on exercise interventions in preclinical models of cancer are favored. To date, there are few animal studies looking at physical activity and cancer that have reported beneficial outcomes [20, 21]. Voluntary wheel running has been reported to inhibit the formation of chemically induced colon [22] and breast cancer in rats [23], skin cancer [24], Panc-1 pancreatic tumors [25], and androgen-independent PC-2 prostate tumors in mice [26]. Zheng et al found that voluntary exercise inhibits the growth of prostate tumors in SCID mouse xenograft model and can enhance apoptosis in PCa tumors [20]. Esser et al., reported that 10 weeks of voluntary running at a rate of >5 km/day delayed PCa incidence and progression compared animals that ran <5 km/day [27].

While exercise interventions may be beneficial in preventing PCa, the majority of Americans fail to meet the recommended guidelines of 30 mins of activity per day or 150 mins per week [28]. This is similarly true with cancer patients, as majority of patients with PCa do not exercise regularly [29–32]. Therefore, the investigation of alternative therapies that act as an exercise mimetic can be doubly beneficial.

Nexrutine is a commercially available herbal extract from the *Phellodendrom amurense*, which is widely used for the treatment of inflammation, gastroenteritis, abdominal pain and diarrhea.[33] The tree is native to Asia and has been reported to contain isoquinoline, alkaloids, phenolic compounds and flavone glycosides. Recently, we have demonstrated that Nexrutine inhibits PCa cells growth implicating a role for Nexrutine in modulating growth factor signaling [34]. Currently, our understanding how Nexrutine compared to other non-toxic, non-pharmaceutical interventions, such as exercise, is missing. To that extent, we conducted a comparative effectiveness study of Nexrutine and exercise on PCa growth in the transgenic adenocarcinoma of mouse prostate (TRAMP) model. We hypothesized that Nexrutine and exercise will have similar inhibitory effect on tumor growth through the modulation of growth and inflammatory signaling. Briefly, the results of this comparative effectiveness study suggest both Nexrutine and exercise inhibit tumor growth with both treatment groups demonstrating significantly fewer high grade tumors compared to the control group.

## Materials and methods

**Fig 1:**
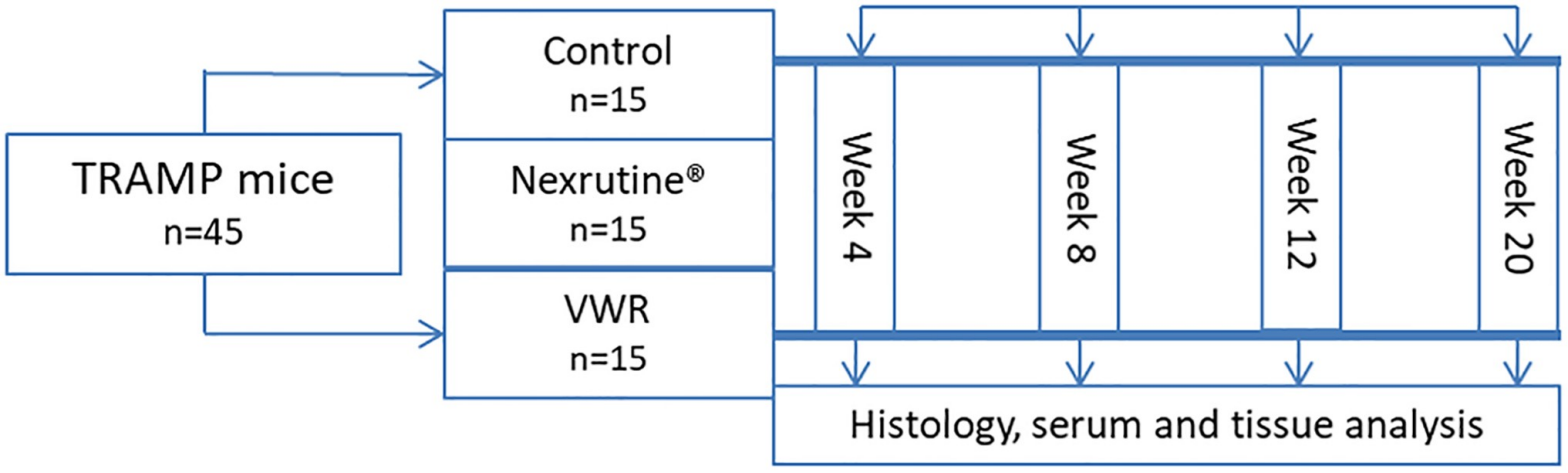
Experimental study design.

The primary aim for this comparative effectiveness study was to examine if Nexrutine can serve as an exercise analog in inhibiting tumorigenesis in the transgenic adenocarcinoma of the mouse prostate (TRAMP) model. Forty-five, 10-week old male TRAMP mice were randomized into one of three groups: Nexrutine alone, exercise alone or control. Three animals from each group were scheduled for sacrifice at weeks 4, 8, and 12, with the remaining mice sacrificed at week 20 (Fig 1).

### Transgenic mouse model

A total of 45, 10-week old male transgenic adenocarcinoma of the mouse prostate (TRAMP) mice, developed by prostate-specific expression of SV40 large T antigen using the rat probasin promoter, were used in this study. TRAMP mice develop prostate tumors with 100% frequency, in progressive stages that facilitates preclinical studies in the prevention, intervention and regression setting as demonstrated by various groups, including ours [34–37]. TRAMP mice with a pure C57BL/6 background were obtained from Jackson Laboratories (Bar Harbor, ME, USA). SV40 gene expression was verified via western blot (Supplementary Information). Mice were maintained in a climate-controlled environment with a 12-hour light-dark cycle with diet and water provided *ad libitum*. At necropsy, animals were examined to determine if there are any gross organ abnormalities. Animal care and handling was in accordance with established humane guidelines and protocols approved by the University of Texas Health Science Center at San Antonio’s Institutional Animal Care and Use Committee.

### Voluntary wheel running

Mice randomized to the exercise group were given continuous access to a running wheel measuring 11.5 cm in diameter, with wheel revolutions monitored continuously by magnetic sensor (Med Associates, Inc., St. Albans, VT, USA). This modality was chosen for the exercise intervention, as opposed to forced exercise modalities (i.e. forced treadmill running) because it is most reflective of natural mouse locomotion and behavior. In addition, forced exercise has the potential to induce a stress response that may alter the outcomes of our study. Voluntary wheel running is characterized by short period of performance against a low load throughout the entire dark cycle. Therefore, the running wheel modality reflects activity that is consistent with exercise behavior inversely correlated with mortality and closely mimics the capabilities of PCa.

### Preparation of Nexrutine diet

Nexrutine was obtained from Interhealth (Benicia, CA, USA). Based on positive results from previously published data, a dose of 600 mg/kg Nexrutine was used [34]. Nexrutine was pelleted into a standard AIM-93G purified roden diet by Dyets, Inc (Bethlehem, PA, USA). This diet consisted of casein (200 g/kg), Deytrose (132 g/kg) cornstarch (397.5 g/kg), surcrose (100 g/kg), cellulose (50 g/kg), soybean oil (70 g/kg), t-butylhydroquinone (0.014 g/kg), satl mix #210050 (35 g/kg), vitamin mix #310025 (10 g/kg), L-cystine (3 g/kg), and choline bitartrate (2.5 g/kg). Quality assurance and irradiation was performed by the manufacturer. Stability of Nexrutine in the diet pellets was evaluated and reported previously [34–37]. Access to Nexrutine pelleted diet was provided ad lib and food consumption was assessed weekly.

### Histology and immunohistochemistry

Genitourinary complex were harvested, weighed and fixed in 10% neutral buffered formalin. Tumors sections were dissected, paraffin embedded, sectioned, placed on poly-lysine slides and stained with hematoxylin and eosin stain to visualize cell nuclei and cytoplasm. Sectioned tumor slides were scored in a blinded fashion using an established grading system for TRAMP mice [35, 38]. Non-cancerous lesions were graded as 1, 2 or 3, indicating normal tissue, low PIN and high PIN, respectively. Grades 4, 5 and 6 are indicative of well-differentiated, moderately differentiated and poorly differentiated cancerous lesions, respectively.

To determine the physiological adaptations within the tumor, 20 week samples were probed with antibody specific to mouse pAkt (Ser473, rabbit monoclonal, 1:50, Cell Signaling, Danver, MA) and p65 NFκB (Ser536, rabbit monoclonal antibody, 1:1000, Cell Signaling, Danver, MA). Secondary and tertiary antibodies were biotinylated and stripped with streptavidin horseradish peroxidase (Biocare 4 plus, Kit, Biocare Medical, Concord, CA). Slides were then reviewed by a pathologist blinded to the treatments. Immunoreactivity was scored based on the percentage of stained cells and graded semi-quantitatively as zero (0% stained cells), 1+ (<10% stained cells), 2+ (10–20% stained cells), 3+ (20–50% stained cells) and 4+ (> 50% cells stained). Images were recorded using a light microscope.

### Cytokine multiplex

Serum collected from mice sacrificed at the 4 week and 20 week time points were assayed in duplicate using a 32 panel Mouse Cytokine/Chemokine Magnetic Bead Panel Immunology Multiplex Assay (EMD Millipore, Billerica, MA) to determine the changes in cytokine profiles. Proteins analyzed include Eotaxin/CCL11, G-CSF, GM-CSF, IFN-γ, IL-1α, IL-1β, IL-2, IL-3, IL-4, IL-5, IL-6, IL-7, IL-9, IL-10, IL-12 (p40), IL-12 (p70), IL-13, IL-15, IL-17, IP-10, KC-like, LIF, LIX, MCP-1, M-CSF, MIG, MIP-1α, MIP-1β, MIP-2, RANTES, TNF-α, VEGF.

### Statistical analysis

One-way analysis of variance (ANOVA) was performed to determine significant differences between treatment groups. Dichotomous measures, such as the presence or absence of palpable tumors were evaluated for treatment group differences using Fisher’s exact tests. Mann-Whitney U-tests were performed for each intervention duration (weeks 4, 8, 12, and 20) comparing tumor grades of Nexrutine treated animals and exercising animals to control mice. Based on previous studies conducted by our group the effect size of Nexrutine is approximately 1.3, which implies a sample size of 15 mice per group completing the project provides greater than 85% power to detect significant changes between groups with a one-sided alpha of 0.05. All statistical testing were two sided with a significance level of 5%. Statistical Package for the Social Sciences (SPSS) (IBM, Armonk, NY) was used to conduct the analysis. GraphPad Prism (La Jolla, CA) was used to develop the graphs.

## Results

### Intervention fidelity

**Fig 2:**
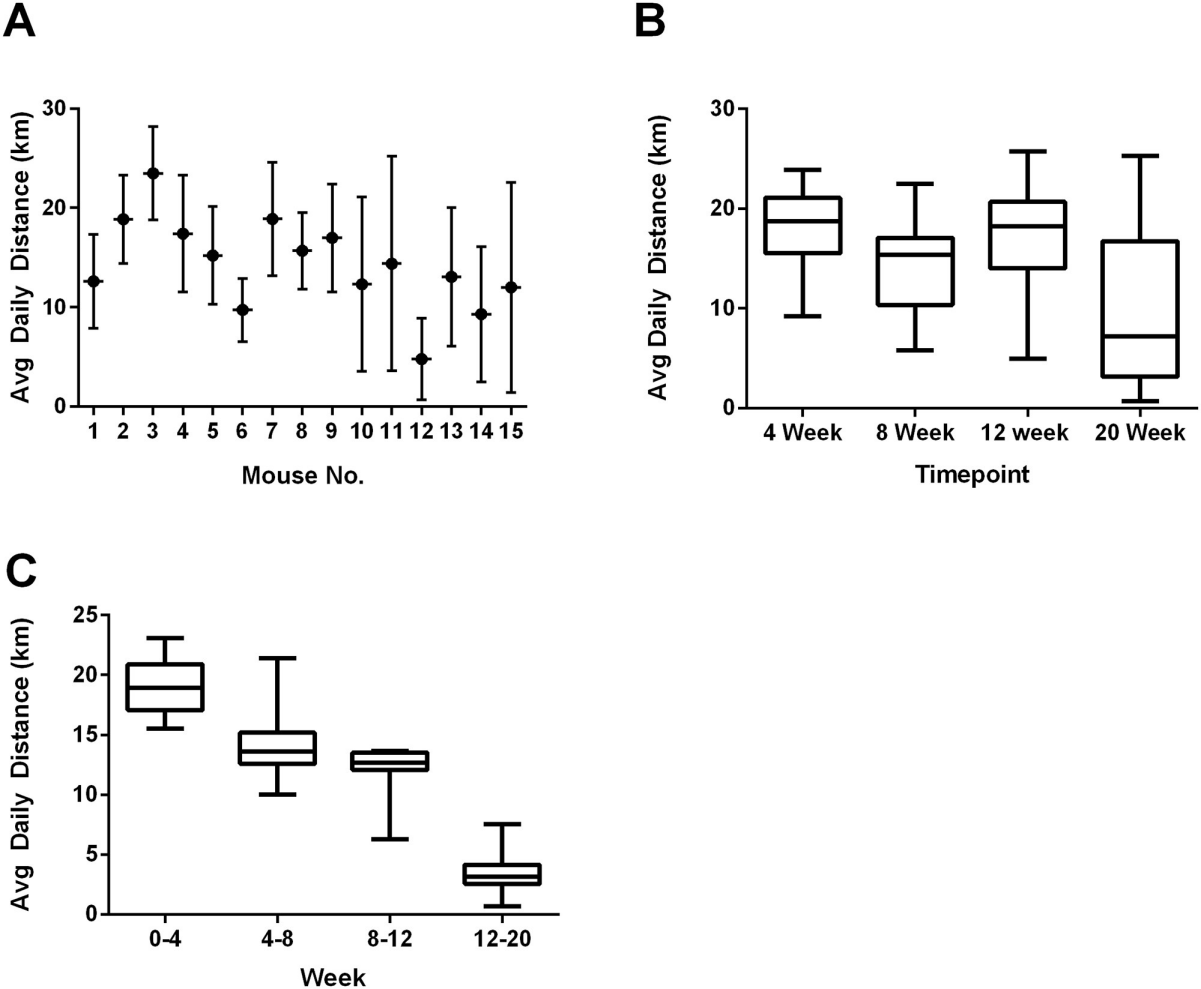
Voluntary wheel running daily variability. This graphical display presents running wheel activity (km/day) as calculated based on the number of revolutions of the running using the wheel manager data analysis software provided (Med Associates, Inc). This data suggests variability in distance ran dependent on the timepoint which the mice were sacrificed. Panel A presents daily distance averages for each mouse in the VWR group. Panel B presents an average daily distance for the mice scarified at each time point (n-3 for 4, 8, and 12 week timepoints; n = 6 for 20 week timepoint). Panel C presents VWR data for all living mice during the weeks 0–4 (n = 15); week 4–8 (n = 12), week 8–12 (n = 9) and week 12–20 (n = 6). This data suggests running distance decreases as the study progresses.

On average, Nexrutine fed mice consumed 18.10 ± 1.28 g of chow per week, equivalent to approximately 15 mg of Nexrutine per day (Table 1). Exercising mice ran 14.31 ± 1.8 km/day. Variability of running distance is presented in Fig 2. Mean body weight changes were analyzed with respect to treatment and time point. No significant differences were observed between groups at the respective time points (4, 8, 12, and 20 weeks) nor was a treatment effect observed. Tumor free body mass was also analyzed by taking the difference between total body mass and GU mass. No significant treatment effect was observed across the 20 week study. The only significant time point difference was seen at 12 weeks (f = 4.982; p = 0.045) with Nexrutine fed mice having less tumor free body mass compared to exercise mice (Nexrutine: 23.7 ± 3.76; Exercise: 30.1 ±1.27; p = 0.04). Since the animals in the Nexrutine group did not demonstrate any significant loss in body mass, these data indicate that Nexrutine is non-toxic in nature (Table 1).

**Table 1:** Animal characteristics.

	Control	Nexrutine^®^	Exercise
Body Mass (g)	33.2 ± 7.28	31.5 ± 3.96	35.2 ± 8.5
GU Mass (g)	4.92 ± 7.79	4.65 ± 5.95	7.18 ± 8.44
GU Free Body Mass (g)	28.28 ± 2.95	27.35 ± 4.24	28.02 ± 2.13
Food Consumption (g/week)	26.06 ± 3.57	18.10 ± 1.28	28.22 ± 2.98

### Nexrutine and exercise suppress palpable tumors in TRAMP mice

Tumors were first palpable in the control group with 27% of control mice demonstrating presence at 14 weeks of age (week 4 of the intervention). Tumor were not palpable in the exercise or Nexrutine group until 5 weeks later (19 week old or Week 9 of the intervention). At 19 weeks of age, 8 of the 9 remaining TRAMP mice in each group presented with palpable tumors. Analysis of this data showed that exercise intervention and dietary intervention with Nexrutine suppressed the occurrence of palpable tumors (p = 0.05, control vs. exercise or Nexrutine). These data support the potential for Nexrutine and exercise to reduce tumor incidence and/or increasing the latency of tumor development.

### Nexrutine and exercise slow prostate tumor progression in TRAMP mice

**Fig 3:**
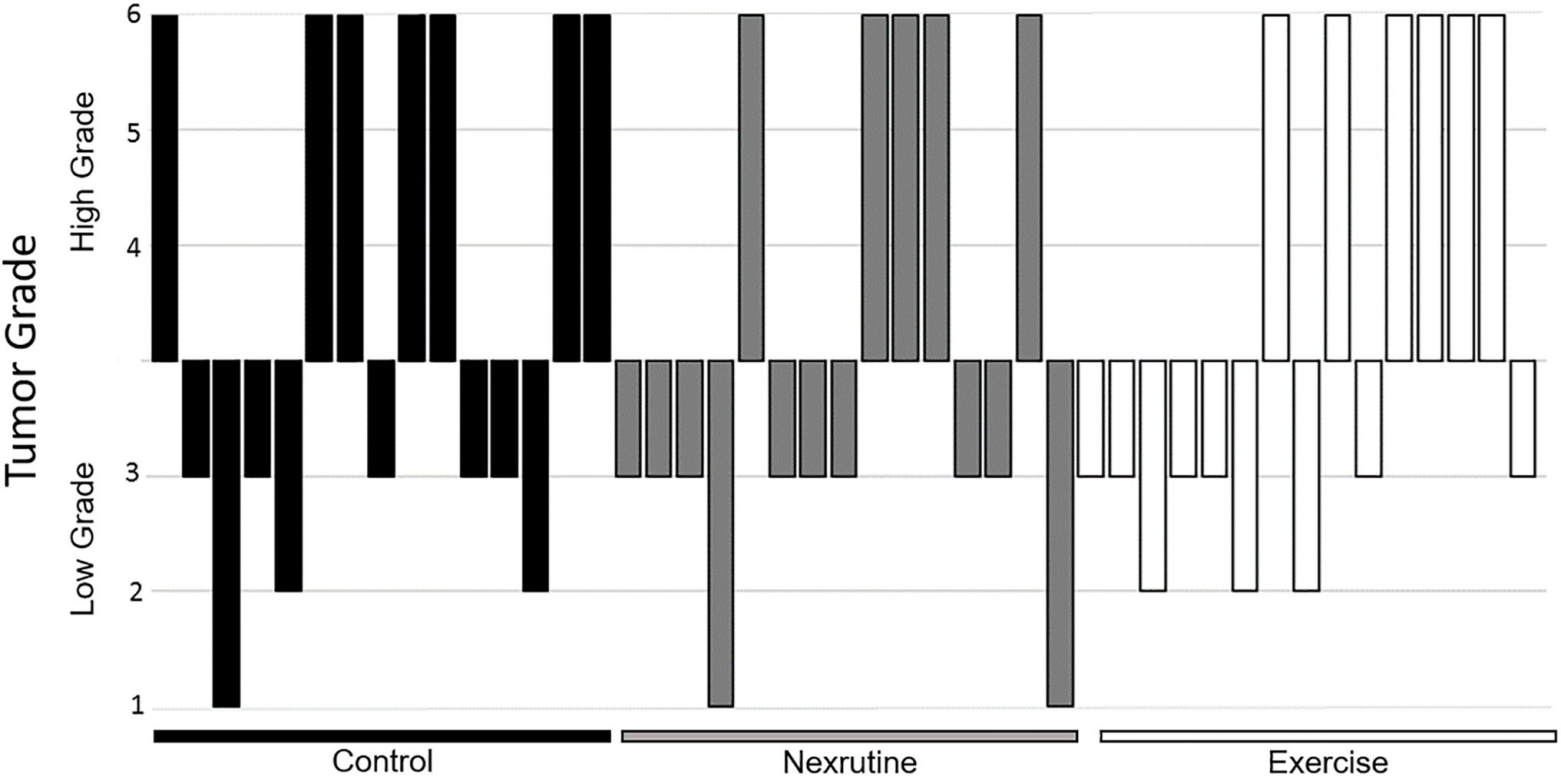
Waterfall Diagram presenting group variability of tumor grades. Individual tumor grades are presented for each group (control, Nexrutine^®^, Exercise). Data is presented as either low grade (scores 1–3) or high grade (scores 4–6).

**Fig 4:**
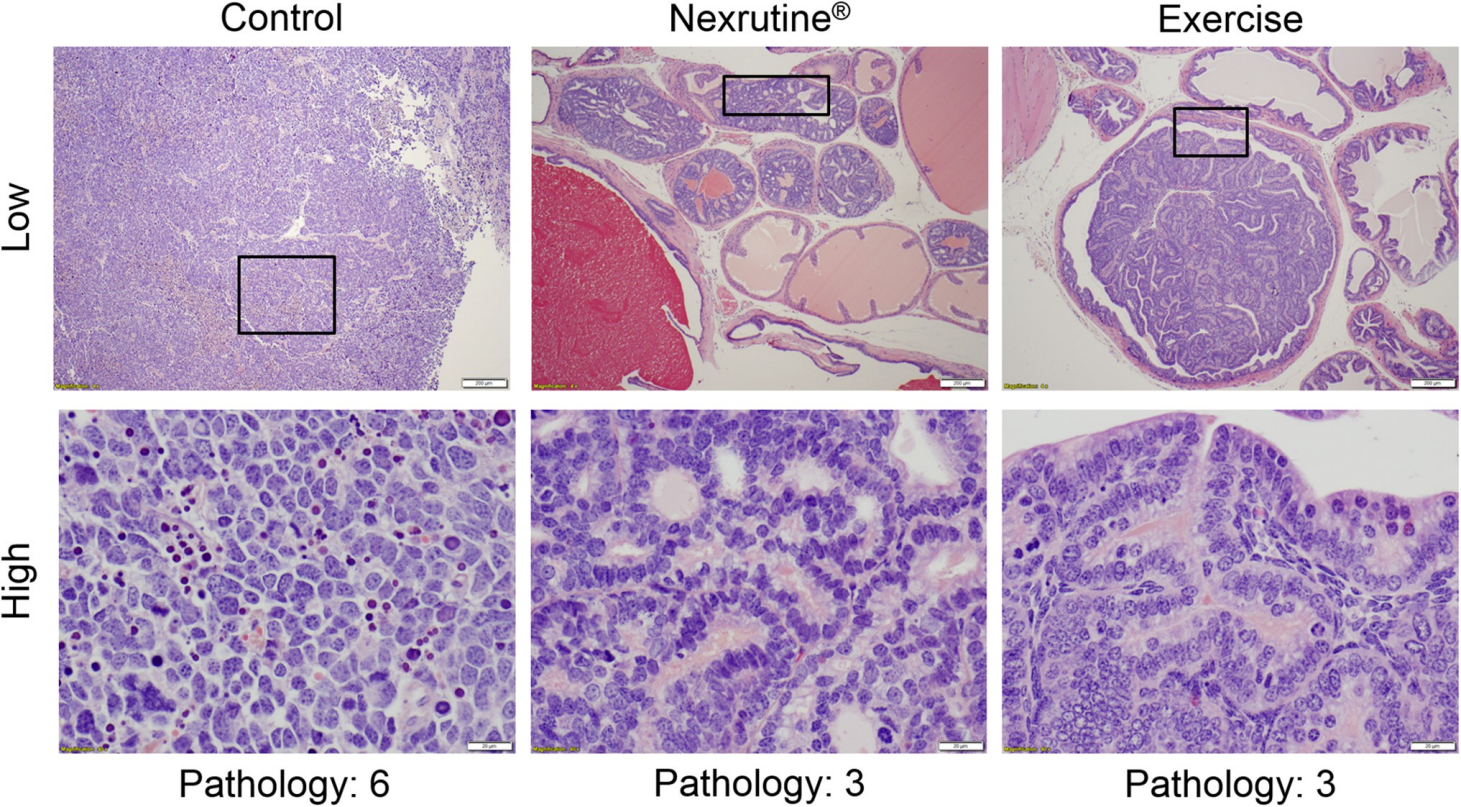
Histology of representative tumors. Prostate lesions were scored in a blinded fashion using an established grading system for TRAMP mice [35, 38]. Non-cancerous lesions were categorized as low grade (graded as 1, 2 or 3), indicating normal tissue, low PIN and high PIN, respectively. Grades 4, 5 and 6 were categorized as high grade, indicative of well-differentiated, moderately differentiated and poorly differentiated cancerous lesions, respectively. Pathology scores were significantly higher in the control group with 71% scoring high compared to Nexrutine^®^ and exercise mice. Representative low magnification (200 μm bar) and high magnification (20 μm bar) images for control, Nexrutine^®^ and exercise mice completing 20 weeks of intervention are presented.

No significant pathological changes were observed as a function of time; therefore, data were pooled for analysis. Contrary to our hypothesis, neither exercise nor Nexrutine were able to significantly reduce GU mass (Table 1). However, exercise and Nexrutine were able to reduce the number of high grade tumors. Specifically, Nexrutine fed mice were observed to have a 60% beneficial effect in protecting against high grade tumors with only 5 of the 15 mice in the group presenting with high grade tumors. Exercise had a 60% beneficial effect with only 6 of the 15 mice having high grade tumors (Fig 3). Histopathological evaluation of the prostate tumor from the 3 study groups is shown in Fig 4. Prostate tumors from control mice show poorly differentiated tumor characterized by variable nuclear shape with no gland formation. In comparison, prostate tumor samples from animals fed Nexrutine or exercising TRAMP mice exhibit pathological features consistent with high grade prostatic intraepithelial neoplasia. While 100% of animals developed tumors (varying stages), Nexrutine fed and exercising TRAMP mice had fewer poorly differentiated tumors compared to controls.

### Nexrutine, but not exercise, reduces pAkt expression in prostate tumors

**Fig 5:**
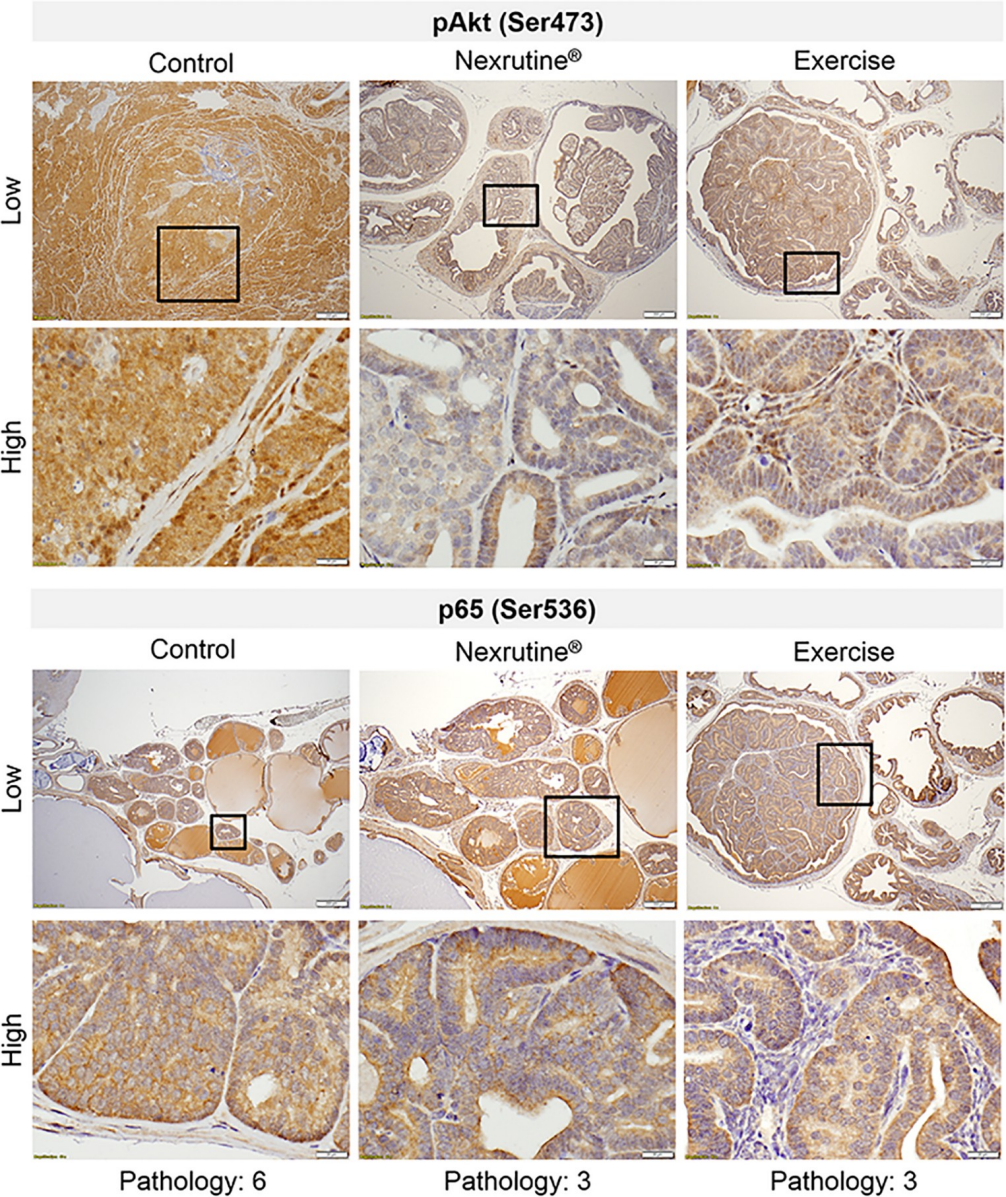
Nexrutine^®^ reduces pAkt expression in prostate tumors. Sectioned prostate tumors from 20 week samples were probed with antibodies specific to pAkt and p65 NFκB and reviewed by a pathologist blinded to the treatments. Immunoreactivity was scored based on the percentage of stained cells and graded semi-quantitatively as zero (0% stained cells), 1+ (< 10% stained cells), 2+ (10–20% stained cells), 3+ (20–50% stained cells) and 4+ (> 50% cells stained). Representative microscopic photos are at low magnification (200 μm bar) and high magnification (20 μm bar) suggesting reduced expression of pAkt indicative of a potential mechanism of action in reducing high grade tumors in Nexrutine^®^ fed mice.

Consistent with previous work by our group, Nexrutine fed TRAMP mice has significantly lower expression of pAKT (Ser473) compared to control (p = 0.04; Fig 5) and exercising TRAMP mice (p = 0.017), respectively. Contrary to our hypothesis, 20 weeks of exercise did not reduce expression of pAkt (Ser473). Additionally, no differences were observed in p65 expression between the 3 study groups.

### Exercise and Nexrutine modulate the cytokine profile of TRAMP mice

**Fig 6:**
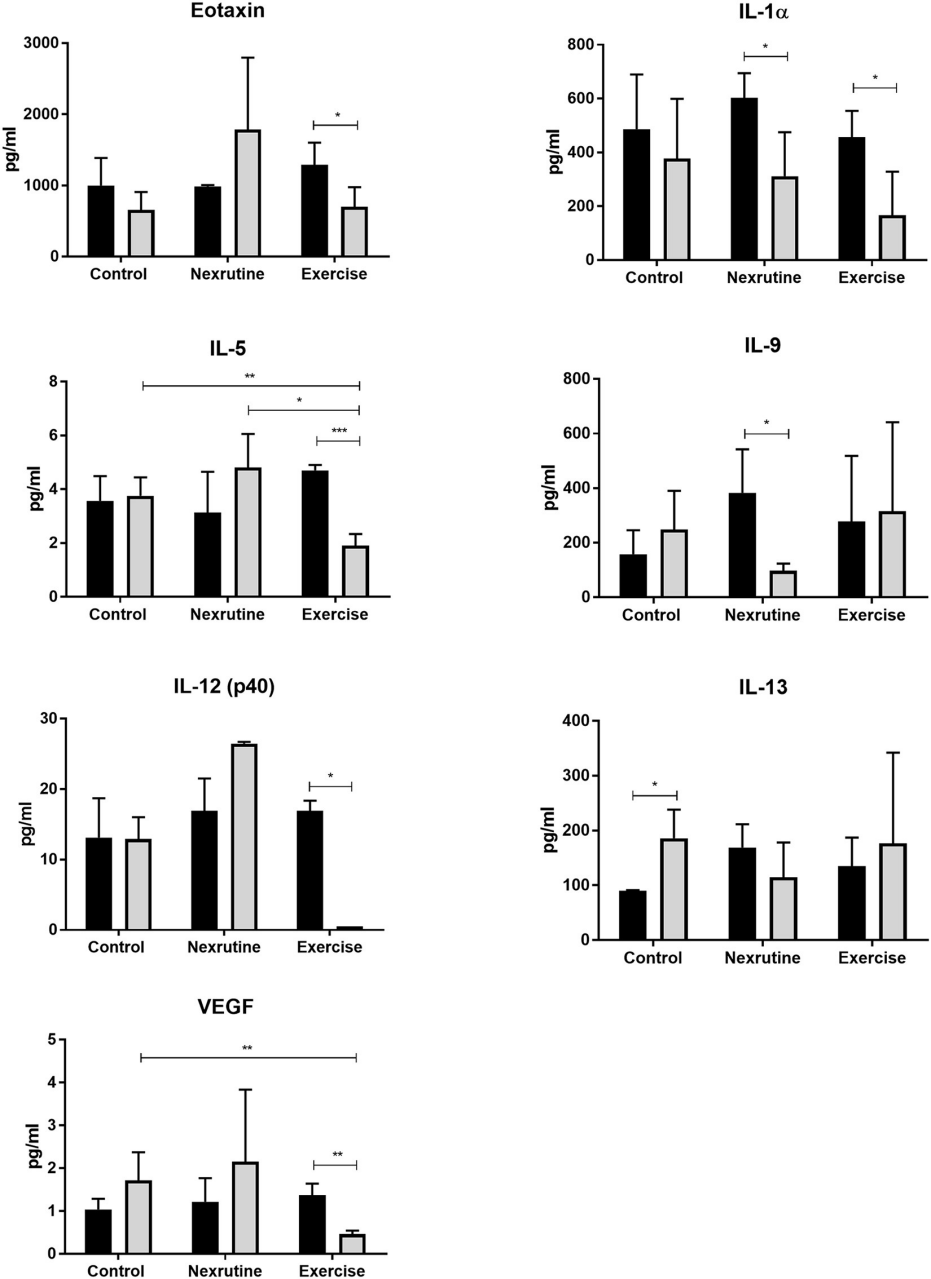
Significant differences observed in cytokines associated with tumor progression. Samples from week 4 (black bars) and week 20 (gray bars) from each group were assayed using a 32 cytokine multiplex. Though there is variability in cytokines that were observed to be significant, both exercise and Nexrutine^®^ similarly reduced concentrations of IL-1α, a cytokine associated with cachexia, anorexia and bone loss. * indicates significant at p<0.05. ** indicates significance at p<0.01. *** indicates significance at p<0.001.

Of the 32 cytokines measured in week 4 and week 20 samples, control mice had borderline significant changes only in IL-13 (p = 0.04; Fig 6). Whereas, exercise resulted in lower concentrations of eotaxin, IL-1α, IL-5, IL-12(p40) and VEGF p<0.05). While there were no differences at baseline, exercise mice had lower IL-5 and VEGF compared to control at the 20 week time point (p<0.05). Nexrutine significantly reduced circulating IL-1α and IL-9 concentrations however, no significant differences were observed when compared to the control group (Fig 6).

## Discussion

The purpose of this study was to compare the effectiveness of exercise and Nexrutine interventions in delaying tumor progression in TRAMP mice. Combining the time points through the 20 week intervention, we found that both exercise and Nexrutine were able to reduce the number of high grade tumors at necropsy. The outcomes in the Nexrutine group support work previously published by our group and others on the antitumorigenic capabilities of this natural product [39–41]. Similarly, the results seen in the exercise group support previous work suggesting exercise has cancer preventative traits. Here we report that the anti-inflammatory effects of exercise may be a mechanism by which exercise exerts its benefits. We also aimed to identify the role of Nexrutine as an exercise analog. Our results represent the first of its kind to report comparing a natural product to exercise. The outcomes of our study suggest that both Nexrutine and exercise can protect against aggressive tumors, thought it appears that they do so through different mechanisms.

It is well established that there is a negative association of PCa with consumption of fruits and vegetables [42]. Nexrutine is a commercially available herbal extract from the *P*. *amurense*, a tree native to Asia widely used for the treatment of inflammation, gastroenteritis, abdominal pain and diarrhea [36]. Recently, our group published a review of the benefits of Nexrutine as a chemopreventative agent [39]. The results of this study provide preliminary evidence that this herbal extract inhibits tumor development similarly to exercise.

The translational potential of Nexrutine was recently demonstrated in a first of its kind study by our group demonstrating the safety of Nexrutine supplementation in a phase I clinical trial [43]. In this study, men diagnosed with PCa received Nexrutine orally (500 mg three times a day) either 1 to 2 months preoperatively or 1 to 2 months prior to or concurrently with radiation therapy. By the end of the neoadjuvant treatment, 81% of the patients had a decline in prostate specific antigen (PSA). More importantly, this study revealed minimal and self-limited toxicities.

The current context of exercise as a chemopreventative intervention is mixed with many studies reporting on the benefits of physical activity [44], but some reporting physical activity is unfavorable to reducing PCa risk [45]. Although the precise biological mechanisms linking physical activity to reduced PCa risk are debated, McTiernan and colleagues suggest the role of reduced inflammation and improved immune function as key contributors [46]. The results of our investigation suggest that aerobic exercise can reduce key cytokines associated with tumor progression.

Growth signaling through Akt has been associated with PCa development. Therefore, altering Akt activity/phosphorylation may inhibit tumor cell proliferation and gene transcription [47]. In this study, exercise reduced high grade tumors; however, exercise did not alter intratumoral pAkt ((Ser473). Standard and colleagues (2009) reported previously that 12 weeks of ad-lib aerobic exercise did not affect Akt gene expression [48]. Exercise is a strong promotor of growth factor signaling [49], which may be counterintuitive as an intervention to reduce tumor mass. However, the reduction in high grade tumors reported in this publication provide valuable rationalization for the use of aerobic exercise as a preventative intervention. Nexrutine on the other hand was able to reduce tumor expression of pAkt (Ser473), supporting previous work by our group [35, 39, 41]. Continued research in the area of growth factor signaling in relation to tumor growth and aggressiveness in the context of aerobic exercise is needed.

Aerobic exercise has been reported to have significant anti-inflammatory effects [46]. This led us to the hypothesis that exercise would exert its effect by modulating cytokine signaling evident by reduced p65 NFκB. This hypothesis was partially supported with lower concentrations of circulating eotaxin, IL-1α, IL-5, IL-12 (p40) and VEGF. Nonetheless, the decreased inflammatory profile in exercise TRAMP mice was not associated with changes in p65 NFκB in the tumor.

The results of our study found that both Nexrutine and exercise reduced circulating concentrations of IL-1α. IL-1α is a regulatory cytokine that can induce the activation of transcription factors and promotes the expression of genes involved in cell survival, proliferation, and angiogenesis [50]. IL-1α has also been implicated in cachexia and anorexia [51, 52]. IL-1α, as well as other cytokines, such as IL-6, IL-11 and TNF-α activate the RANK pathway that controls bone remodeling [53]. While bone density was not measured in this study, our group has reported previously that Nexrutine preserves bone density in TRAMP mice [37]. In cases of advanced cancers, bone mineral density loss is often observed and may be mediated via IL-1α. Therefore, a reduction in IL-1α concentrations could suppress osteoclastogenesis and osteoclast activity [54]. Others have also reported exercise prevents bone loss PCa patients [55]. Future research is needed to elucidate the mechanism by which exercise and Nexrutine decrease IL-1α concentrations and the role of IL-1a on cachexia and bone loss seen with cancer.

The decreased circulating concentrations of cytokines provides insight to the benefits of exercise in suppressing tumor progression. Eotaxin is of significant interest as an exercise target due to its role as a potential prognostic biomarker for PCa [56]. Even though little is known regarding the role of eotaxin in tumor pathology, eotaxin is known to activate matrix metalloproteinases that are implicated in the in PCa progression [57]. Others have also reported that eotaxin is able to promote cell migration in vitro and induce angiogenesis in vivo [58]. This reduction in eotaxin may account for the reduced VEGF concentration observed in our study.

In our study, exercise resulted in lower circulating VEGF concentrations. The suppression of VEGF of significance because of the vasculature remodeling that occurs in solid tumors. Tumor blood vessels are structurally and functionally abnormal [59]. Greater than 50% of tumor vessels are non-functional [60]. Reducing VEGF has the potential of inhibiting vessel sprouting and normalize tumor vasculature [61]. VEGF can been implicated in the proliferation, survival, adhesion, migration and chemotaxis of tumor cells [62]. Thus, reducing VEGF will likely delay tumor progression as seen in this study. Further, the discovery of the multiple isoforms of VEGF raise the possibility that they each have different functions [62–64].

Our study isn’t without limitations. First, the study design with multiple timepoint sacrifices decreased statistical power for our study. Based on previous studies conducted by our group, a sample size of 15 mice per group provides 80% power to detect significance changes between groups. Additionally, the exercise modality of leads to great variability in exercise durations. Future research should consider the variance between forced exercise compared to voluntary exercise interventions. Similarly, consumption of Nexrutine through the diet is variable as well. Alternatives would include daily gavage or injection of suspended Nexrutine. While it may provide consistent dosing of Nexrutine, these protocols may also stress animals which may impact outcomes measures.

The results of our study indicate that both exercise and Nexrutine can independently reduce tumor aggressiveness. The mechanisms by which these interventions exert their positive effects different, suggesting that aerobic exercise prevents tumor aggressiveness by modulating circulating cytokines while Nexrutine exerts is benefit by reducing intratumoral growth factor signaling. Tumor growth and proliferation are managed through a number of different signaling pathways. The results of our study suggest that future research considering combining the two interventions to investigate an additive/synergestic effect that may target tumor grown through multiple mechanistic pathways.

In conclusion, the results of our study suggest that the natural produce Nexrutine and exercise similarly reduce PCa risk and severity. Future research is necessary to identify commonalities and differences in the mechanisms by which exercise and Nexrutine exert this beneficial effect on prostate cancer.

## Supporting information

S1 Table Study database. (XLSX)

S2 Table Absolute values of cytokine concentrations with means ± standard deviations. (XLSX)

S1 Fig SV40 transgene expression PCR protocol. (JPEG)

S2 Fig SV40 expression western blot results. The ladder in lane 1 is New England BioLabs Quick-Load 100 bp DNA Ladder (catalog number N0467). Lanes 2–5 represent samples from animals carrying the transgene. Lanes 6–8 are from animals that are not carrying the transgene.
(JPG)

## Abbreviations

ANOVA: analysis of variance
g/kg: grams per kilogram
G-CSF: granulocyte-colony stimulating factor
GM-CSF: granulocyte-macrophage colony-stimulating factor
GU: genitourinary
IFN-y: interferon-gamma
IL: interleukin
IP-10: Interferon gamma-induced protein 10
KC-like: chemokine (C-X-C- motif) ligand
km/day: kilometers per day
LIF: leukemia inhibitory factor
LIX: lipopolysaccharide-induced C-X-C motif chemokine
MCP-1: monocyte chemoattractant protein 1
M-CSF: macrophage colony-stimulating factor
mg/kg: miligram per kilogram
MIG: monokine induced by gamma interferon
mins: minutes
MIP: macrophage inflammatory protein
NFκB: nuclear factor kappa-light-chain-enhancer of activated B cells
pAkt: phosphorolated Akt (protein kinase B)
Panc-1: pancreatic cancer model
PC-2: prostate cancer -2 model
PCa: prostate cancer
PIN: prostatic intraepithelial neoplasia
PSA: prostate specific antigen
RANTES: Regulated on Activation, Normal T Cell Expressed and Secreted
SCID: severe combined immunodeficiency
SPSS: Statistical Package for the Social Sciences
SV40: simian virus 40
TNF-α: tumor necrosis factor - alpha
TRAMP: transgenic adenocarcinoma of the mouse prostate
VEGF: vascular endothelial growth factor
VWR: voluntary wheel running
