# Supplementary figures and images for: Nexrutine and exercise similarly prevent high grade prostate tumors in transgenic mouse model
Source: PLoS One. 2019 Dec 19;14(12):e0226187. doi: 10.1371/journal.pone.0226187 (PMC6922346; doi:10.1371/journal.pone.0226187)

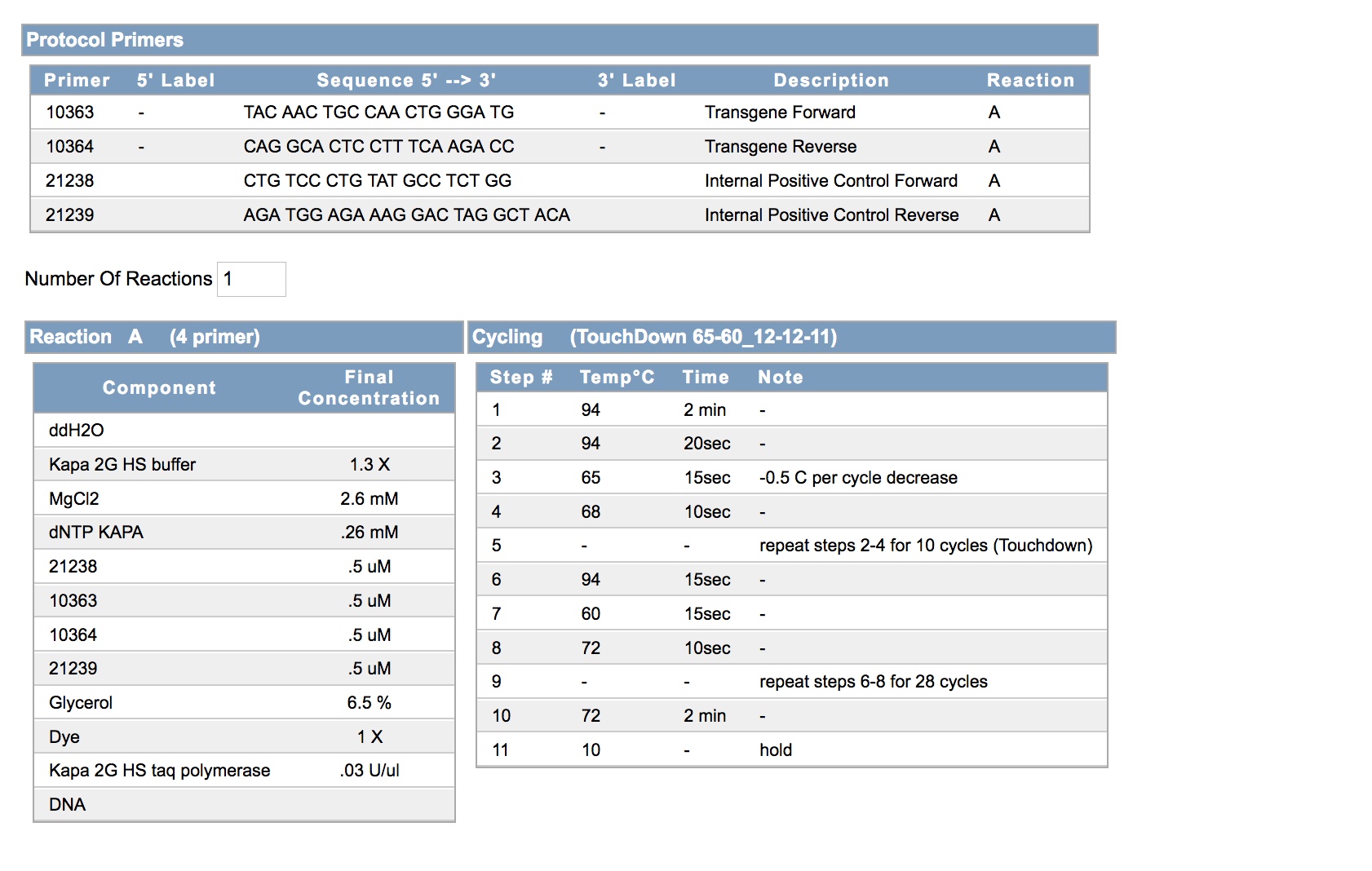

Supplement: S1 Fig — (JPEG) [file pone.0226187.s003.jpeg]

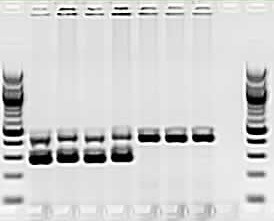

Supplement: S2 Fig — The ladder in lane 1 is New England BioLabs Quick-Load 100 bp DNA Ladder (catalog number N0467). Lanes 2–5 represent samples from animals carrying the transgene. Lanes 6–8 are from animals that are not carrying the transgene. (JPG) [file pone.0226187.s004.jpg]
